# The role of oxidative stress and antioxidant therapy in cisplatin neurotoxicity: preclinical evidence in the last decade

**DOI:** 10.1007/s00204-026-04398-9

**Published:** 2026-04-28

**Authors:** Neife Aparecida Guinaim dos Santos, Júlia Maiara dos Santos, Antônio Cardozo dos Santos

**Affiliations:** Departamento de Análises Clínicas, Toxicológicas e Bromatológicas, Faculdade de Ciências Farmacêuticas de Ribeirão Preto, Universidade de São Paulo, USP, Avenida do Café s/n, Ribeirão Preto, 14040-903 São Paulo Brazil

**Keywords:** Cisplatin, Neurotoxicity, Neuroprotection, Oxidative stress, Antioxidants, Neuroinflammation

## Abstract

Cisplatin remains one of the most effective chemotherapeutic agents for the treatment of several tumors. However, its use is limited by dose-dependent and cumulative neurotoxicity, which affects the peripheral nervous system, hypothalamus, prefrontal cortex, cerebellum, retina and optic nerve. Currently, there is no neuroprotective strategy against cisplatin-induced neurotoxicity. Studies in the last decade have consolidated the role of oxidative stress as a central molecular event in cisplatin-induced neurotoxicity. A wide array of antioxidant-based strategies from natural compounds to repurposed drugs has been investigated in animal models as neuroprotective agents. In general, these compounds act directly through free radical scavenging, or indirectly, by activating the Nrf2 pathway, which induces the expression of antioxidant defense enzymes. This review approaches the role of oxidative stress in preclinical studies on cisplatin-induced neurotoxicity from the last decade, and evaluates the most promising antioxidant interventions, with the focus on cognitive impairment, peripheral neuropathy, ocular toxicity, anxiety and depression. The link between cisplatin neurotoxicity and neurodegenerative diseases as well as the emerging novel therapeutic strategies to replace cisplatin chemotherapy are discussed. The need for future studies in tumor-bearing animal models to investigate interferences with the antitumor efficacy of cisplatin is pointed out as a critical requirement for clinical translation.

## The importance of cisplatin: present and future

Cisplatin (cis-diamminedichloroplatinum II) is one of the most effective chemotherapeutic agents, and it is used, alone or associated with other agents, in the treatment of a variety of tumors, including testicular cancer, ovarian germ cell tumors, epithelial ovarian cancer, head and neck cancer, bladder cancer, endometrial cancer, cervical cancer, non-small cell lung cancer and malignant melanoma (Ferreira et al. 2016; Rose et al. 2024). It is estimated that 50% of cancer patients in the United States are treated with cisplatin either alone, combined with other chemotherapeutic agents, radiation (concurrent chemo-radiation therapy, CCRT) or surgery (Ghosh 2019; Kumar et al. 2018). Dose-limiting toxicities (Elmorsy et al. 2024; Santos et al. 2020) and intrinsic or acquired drug resistance are the major challenges in cisplatin-based anticancer therapy (Rocha et al. 2018). Among the toxicities, neurotoxicity, ototoxicity and nephrotoxicity are the major concerns (dos Santos et al. 2012; Santos et al. 2019, 2020). Cisplatin is the most neurotoxic platinum drug in clinical practice. Cisplatin-induced neurotoxicity is dose-dependent and cumulative (Ferreira et al. 2016; Frisina et al. 2016). Cisplatin-induced toxicities hinder dose escalation, and therefore, limit the therapeutic benefits of cisplatin chemotherapy (for review, see dos Santos et al. 2012; Santos et al. 2020). Consequently, dose reduction or treatment withdrawal is often necessary, as current clinical management of the adverse effects is limited to pain control and is not able to prevent or reverse the underlying neuropathy (Avan et al. 2015; Cetinkaya-Fisgin et al. 2020). Saline hydration, short hydration (with magnesium) and osmotic diuresis (mannitol) have been clinically employed to minimize the nephrotoxicity induced by cisplatin (Ohshima et al. 2025). However, there are no effective prevention or therapeutic measures approved by the FDA to protect against the neurotoxicity induced by cisplatin (Wellenberg et al. 2021).

The efficacy of cisplatin is limited not only by its inherent toxicity but also by the frequent onset of drug resistance. To overcome cisplatin resistance and toxicities, potential alternative treatments have emerged and they are based on immunotherapy (Guidi et al. 2018), molecularly targeted agents (Kumar et al. 2018), hormonal therapy (Abraham and Staffurth 2020), gene therapy (Rangel-Sosa et al. 2017), photodynamic therapy (Agostinis et al. 2011) and nanoparticles-based cisplatin therapy (Babu et al. 2015).

These novel therapies, such as targeted therapy or immunotherapy, might not replace the use of cisplatin in the future, but instead, they will probably be used as adjuvants in combination with cisplatin-based chemotherapy to increase the efficacy and reduce cisplatin-induced toxicities and resistance (Chen and Chang 2019; Gadgeel 2017; Kroon et al. 2019; Scherpereel et al. 2018). For instance, combination of cisplatin with immune checkpoint blockers (such as PD-1/PD-L1 or CTLA4 inhibitors), lipid metabolism disruptors (like FASN inhibitors and SCD inhibitors) and nanoparticle-delivery systems have been proposed to enhance the anti-tumor efficacy of cisplatin (Li et al. 2025). Recently, a D-alpha-tocopherol polyethylene glycol succinate conjugated to hyaluronic acid copolymer (TSHA) was developed. TSHA was designed to specifically target the CD44 protein, which is overexpressed in non-small cell lung cancer (NSCLC) cells, thereby selectively accumulating in tumor tissue. According to the authors, TSHA represents a therapeutic alternative to cisplatin for the treatment of NSCLC, as it achieves antitumor efficacy equivalent to that of cisplatin without causing nephrotoxicity, neurotoxicity or myelosuppression (Chen et al. 2025). These findings are promising for advancing cancer treatment; however, extensive research is required to confirm the potential of this copolymer to replace such a well-established cancer treatment as cisplatin. Despite the advances in targeted and immunogenic therapy, cisplatin has remained the most successful anticancer agent since its FDA approval in 1978 (Chen and Chang 2019; Mariconda et al. 2025), and continues to be the gold standard treatment for solid tumors (Koumaki et al. 2025).

## Reactive oxygen species: the good and the bad stress

Reactive oxygen species (ROS) are molecules that contain oxygen and are highly chemically reactive. The physiological level of reactive oxygen species is termed oxidative eustress, or “good stress” (Jomova et al. 2023). Maintenance of a physiological level of ROS is essential for regulating vital biological processes through redox signaling (Holmstrom and Finkel 2014). ROS are a byproduct of normal metabolism and have roles in cell signaling and homeostasis (Deavall et al. 2012). Minor fluctuations in the concentration of these oxidants play a role in intracellular signaling. Maintaining redox homeostasis is a constant, active process. This state is termed “homeodynamics” to reflect the dynamic process to sustain low to mild levels of oxidants and the regulation of various biochemical processes (Sies 2021).

There are two types of ROS: (1) free radicals, which are unstable, short-lived and very reactive molecules with unpaired electrons (e.g., superoxide anion, O2**·** − and hydroxyl radical, HO**·**, and (2) non-radical species (oxidants), which do not have unpaired electrons but are still chemically reactive (e.g., hydrogen peroxide, H_2_O_2_). Both types can interact readily with other molecules in biological systems, acting as signaling molecules or causing oxidative stress (Jomova et al. 2023). Radicals are more reactive and less stable than non-radicals; however non-radical molecules can be easily converted into free radicals by reactions in the living organisms (Ahmad 2024). Hydrogen peroxide (H_2_O_2_), for example, reacts with ferrous ions, producing hydroxyl radicals, which are highly reactive oxygen species (Fenton reaction: Fe^2+^ + H_2_O_2_ → Fe^3+^+ OH· + OH⁻). Hydroxyl radicals might also be produced via Haber–Weiss reaction, through the reaction between superoxide (O_2_^−^) and H_2_O_2_ (O_2_^-^**·** + H^+^ + H_2_O_2_ → O_2_ + OH**·** + H_2_O) (Fatima-Shad and Das 2023).

Oxidative stress was first defined by Helmut Sies in 1985 as “a disturbance in the prooxidant-antioxidant balance in favor of the former, leading to potential damage” (Sies 1985). A commonly used parameter to quantify this state is the redox balance of plasma glutathione ratio (GSH/GSSG). This balance is observed to become more oxidized with advancing age, exposure to chemotherapy, and in chronic diseases such as type 2 diabetes or cardiovascular disease. However, this definition is limiting, considering that cellular signaling and control processes are mediated by specific redox pathways, whose activity is not always directly correlated with the overall GSH/GSSG ratio, as implied by the classic definition. The total GSH/GSSG ratio might remain unaltered, while certain specific redox signaling pathways are disrupted. Therefore, considering the mechanistic perspective, oxidative stress has been more precisely characterized as a disruption of redox signaling and control (Jones 2006). Since then, the definition has been expanded to include molecular consequences into the previous definitions: Oxidative stress is an imbalance between oxidants and antioxidants in favor of the oxidants, leading to disruption of redox signaling and control, and/or molecular damage (Sies 2019; Sies 2007).

There are endogenous mechanisms that regulate cellular levels of ROS, as their excess can cause damage to key cellular components. Uncontrolled increases in these reactive oxygen species lead to chain reactions with proteins, lipids, polysaccharides, and DNA (Droge 2002). This damage impairs energy metabolism, cell signaling, cycle control, transport and biological activities, causing inflammation and cellular dysfunction (Ahmad 2024). The damage induced by oxidative stress is observed in numerous chronic diseases and in many drug-induced toxicities, including cisplatin-induced toxicities (Alhowail 2025; Deavall et al. 2012).

## Chemotherapy and the aging brain: oxidative stress as a link between cisplatin-induced neurotoxicity and neurodegeneration

A substantial body of evidence implicates oxidative stress in the pathogenesis of many neurological disorders. Brain is highly susceptible to oxidative stress due to its high oxygen consumption, high lipid content, low antioxidant defense and low regenerative capacity; therefore, oxidative stress severely impacts the functions of the central nervous system (CNS). Oxidative stress is involved in neurodegenerative disorders such as Alzheimer´s, Huntington and Parkinson diseases, besides neuropsychiatric disorders such as anxiety and depression (Lee et al. 2020; Salim 2017). Additionally, numerous neurotoxins and drugs induce neurotoxicity through disruption of mitochondrial metabolism and inhibition of antioxidant enzymes, which leads to oxidative stress (Chtourou et al. 2015; Deavall et al. 2012; Wang et al. 2025). For instance, cisplatin causes damage to both neuronal and non-neuronal mitochondria. This damage leads to increased production of reactive oxygen species (ROS) and decreased antioxidant defenses, including reduced glutathione (GSH), and the enzymes superoxide dismutase (SOD), catalase, glutathione peroxidase, and glutathione reductase (McDonald and Windebank 2002; Srivastava et al. 2010). Oxidative damage, mitochondrial dysfunction, and altered energy metabolism are directly implicated in the development of cisplatin-induced neurotoxicity (Maccio and Madeddu 2013). Besides that, these mechanisms are related to the immunoinflammatory response associated with neurodegenerative diseases and epilepsy (Mani et al. 2025).

Although several molecular mechanisms by which cisplatin induces neurotoxicity have been proposed, the exact mechanism remains uncertain (Aziz et al. 2026). Studies in the last decade have consolidated the role of oxidative stress as a central molecular event in cisplatin-induced neurotoxicity (Mani et al. 2025). Cisplatin promotes the generation of reactive oxygen species (ROS) leading to lipid peroxidation, DNA damage, mitochondrial dysfunction, and neuronal apoptosis. The imbalance between oxidant production and endogenous antioxidant defense systems—including glutathione, superoxide dismutase, and catalase—exacerbates neuronal vulnerability (Mani et al. 2025). Cisplatin also causes activation of NF-κB which leads to increased level of pro-inflammatory cytokines like TNF-α, IL-6, and IL-8 and activation of microglial cells, leading to neuronal inflammation and apoptosis (Jangra et al. 2016). Neurotoxicity processes might result from a complex crosstalk of events in which oxidative stress plays a key role, mostly as the initiator of a pathogenic cascade including mitochondrial dysfunction, ER stress, DNA damage, neuroinflammation and apoptosis (Mani et al. 2025). Several hypotheses connecting inflammation and oxidative stress with neurotoxicity are now emerging (Gupta et al. 2022). Oxidative stress and neuroinflammation are distinct, but interconnected processes that influence each other. Mitochondrial impairment and oxidative stress induce microglial activation and inflammatory reactions, and damage synapses and neurons in the CNS (Khandaker et al. 2017; Mondelli et al. 2017). These events have been associated with neurodegenerative diseases and aging (Alhowail 2025). Inflammatory cells produce oxidative stress by increasing the generation of reactive oxygen species (ROS), which in turn promote the production of pro-inflammatory molecules. In this way, neuroinflammation and oxidative stress reinforce each other, intensifying damage within the nervous system. When the redox state is balanced, the inflammatory response is protective; however, oxidative stress disrupts this balance, leading to neuroinflammation in the CNS. Therefore, the inhibition of either oxidative stress or neuroinflammation suppresses the other and, consequently, minimizes neurological damage (Teleanu et al. 2022). There is evidence that cisplatin crosses the blood–brain barrier and causes oxidative stress, mitochondrial dysfunction and neuroinflammation in several brain areas resulting in cognitive impairment (Hagiwara et al. 2023). Accordingly, there is evidence that cisplatin impairs synaptic plasticity and accelerates the biological aging process, by a mechanism that includes oxidative stress, mitochondrial dysfunction, impaired neurogenesis, and neuroinflammation; these events are also associated with increased anxiety and neurocognitive decline (Oliveros et al. 2023). Cisplatin induces both central (Mahmoud Janloo et al. 2024) and peripheral neuropathy, being the latest, more prevalent (Yasar et al. 2019).

One specific consequence of oxidative stress is oxidative DNA damage. It has been demonstrated that the inhibition of enzymes that repair oxidative DNA damage (OGG1 Glycosylase and APE1 Endonuclease) exacerbates the neurotoxicity of cisplatin. Besides being involved in cisplatin-induced neurotoxicity, oxidative DNA damage has a role in the pathogenesis of neurodegenerative diseases, including Alzheimer’s disease (Behrouzi et al. 2022a, 2022b; Deavall et al. 2012). The functional importance of these interconnected mechanisms is supported by preclinical studies showing that antioxidant therapy (Keeney et al. 2018; Lomeli et al. 2017) can alleviate cognitive deficits and neuropathological features in rodent models of chemotherapy-induced cognitive impairment (CICI). These findings highlight the central role of oxidative stress, DNA damage, and mitochondrial dysfunction in the development of CICI. Therapeutic strategies that protect mitochondrial activity and reduce ROS and neuroinflammation are promising for managing neuropathologies and cisplatin induced-neurotoxicity (Ashok et al. 2022; Jangra et al. 2016; Mostafa et al. 2025).

In summary, oxidative stress, DNA damage, mitochondrial dysfunction and neuroinflammation are key biological pathways common to both neurodegenerative diseases and chemotherapy-induced cognitive impairment (Torre et al. 2025). Based on these premises, it could be hypothesized that cisplatin chemotherapy might increase oxidative damage and neuroinflammation in AD patients, that AD patients might be more susceptible to cisplatin-induced oxidative stress and neuroinflammation, or that a synergistic interaction occurs. However, these hypotheses need thorough investigation.

## The role of antioxidant effect in cisplatin-induced neurotoxicity: what research has demonstrated in the last 10 years

This review approaches the neurotoxicity of cisplatin, and the role of oxidative stress in its development and in the protection against it. In order to provide the necessary background and concepts, we searched PubMed database for indexed articles published in English from inception to current date. For updated findings, we searched PubMed database for indexed articles published in English in the last decade (January 2016–March 2026).

Cisplatin affects the peripheral sensory nervous system, hippocampal and cerebellar regions, prefrontal cortex, retinal ganglion cells and optic nerve. The main manifestations of cisplatin-induced neurotoxicity are: cognitive dysfunction /memory and learning impairment (chemobrain); peripheral neuropathy; retinopathy/optic nerve damage and neurobehavioral changes (anxiety/depression) (Alhowail 2025; Cecati et al. 2026; Eldemerdash et al. 2025; Elkattawy et al. 2026; Fidelis et al. 2025; Goel et al. 2025b, a; Mahmoud Janloo et al. 2024; Martínez-Martel and Pol 2023).

**Fig. 1:**
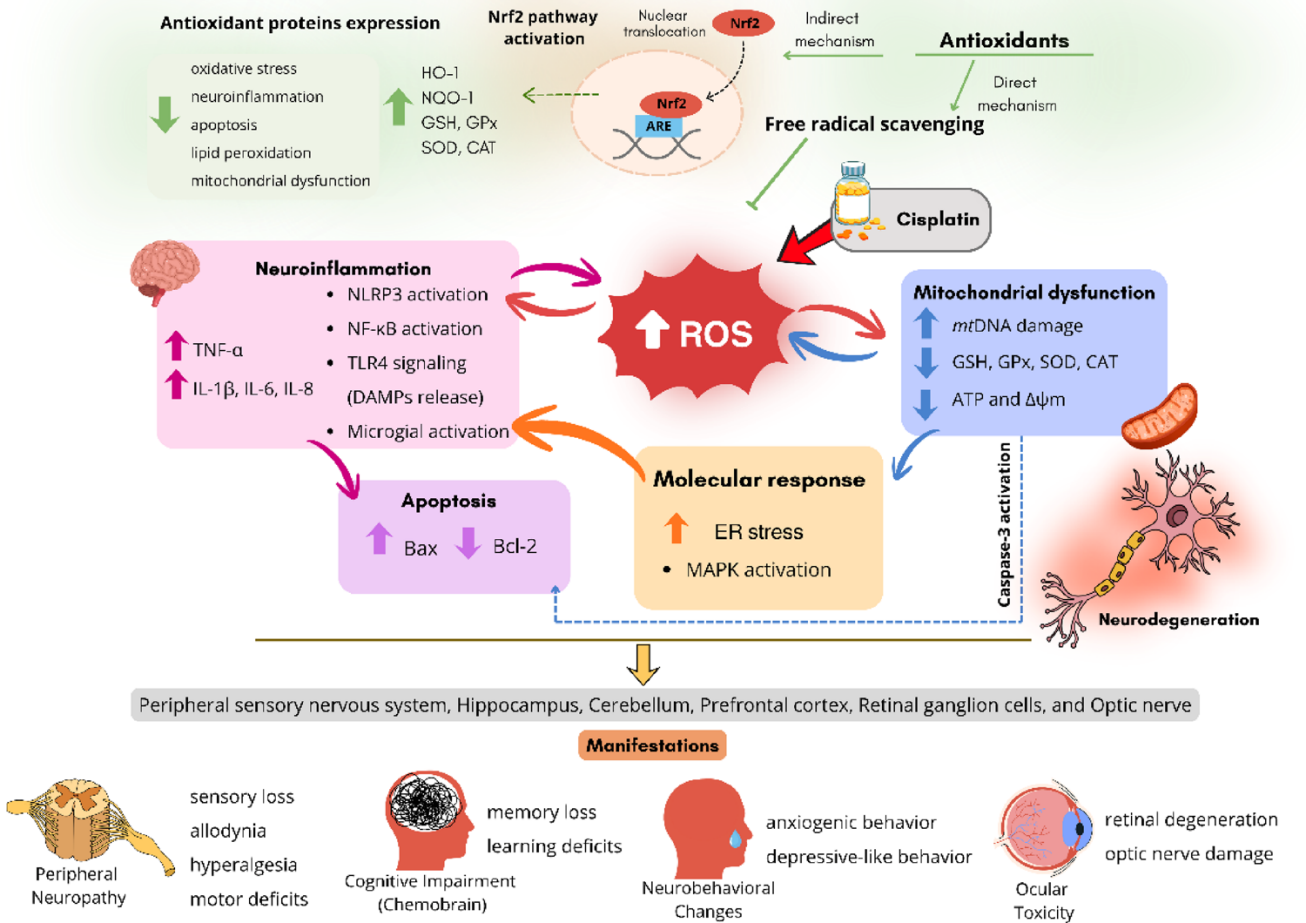
Interconnected pathways of cisplatin-induced neurotoxicity: mechanisms, pathological consequences, and antioxidant neuroprotection. Schematic representation of the pathogenic cascade and amplification loops underlying cisplatin-induced neurotoxicity. Cisplatin triggers the overproduction of reactive oxygen species (ROS), leading to oxidative stress. This initiates a cascade of interconnected events, including mitochondrial dysfunction, DNA damage, endoplasmic reticulum (ER) stress, and activation of the NLRP3 inflammasome, NF-κB pathway, cytokines and microglia. These events collectively promote neuroinflammation and neuronal apoptosis (caspase-3 activation, increased Bax/Bcl-2 ratio). The resulting damage affects different regions of the peripheral and central nervous system, manifesting as peripheral neuropathy, cognitive impairment, retinal and optic nerve damage, and anxiety/depressive-like behaviors. Agents with antioxidant properties (e.g., Nrf2 activators, free radical scavengers) can interrupt this cascade by restoring redox balance and reducing neuroinflammation

In the last decade, several antioxidant strategies have been investigated in pre-clinical studies as potential candidates for neuroprotection against the neurotoxicity induced by cisplatin in different targets. In this review, we summarized (Tables 1, 2, 3 and 4 and Fig. 1) and discussed the antioxidant effects and mechanisms reported in these studies.

**Table 1:** Effects of antioxidant intervention on cisplatin-induced retinal and optic nerve toxicity

Effect of cisplatin	Agent	Neuroprotective effects	Model	References
*Redox (eye tissues)*: ↑ MDA and GSH; ↓ GPx, SOD, and CAT *Histological*: ↑ thickness; ↑ deterioration (retina); ↑ edema; ↑ deterioration (cornea)	Hesperidin	*Redox (eye tissues)*: ↓ MDA and GSH; ↑ GPx, SOD, and CAT *Histological*: ↓ thickness; ↓ deterioration; ↓ edema; ↓ deterioration	Male Sprague–Dawley rats	(Polat et al. 2016)
*Redox (optic nerve tissues)*: ↑ MDA and TOS; ↓ tGSH and TAS *Inflammation (optic nerve tissues)*: ↑ TNF-α and NF-κB *Histological (optic nerve)*: ↑ hemorrhage; ↑ degeneration	Pycnogenol	*Redox (optic nerve tissues)*: ↓ MDA and TOS; ↑ tGSH and TAS *Inflammation (optic nerve tissues)*: ↓ TNF-α and NF-κB *Histological (optic nerve)*: ↓ hemorrhage; ↓ degeneration	Male albino Wistar rats	(Icel et al. 2018)
*Redox (serum)*: ↑ MDA; ↓ tGSH *Inflammation (serum)*: ↑ TNF-α and IL-1β *Histological (retina)*: ↑ edema; ↑ degeneration; ↑ detachment of retinal pigment epithelium and photoreceptor layer	Lutein	*Redox (serum)*: ↓ MDA; ↑ tGSH *Inflammation (serum)*: ↓ TNF-α and IL-1β *Histological (retina)*: ↓ edema; ↓ degeneration; ↓ detachment of retinal pigment epithelium and photoreceptor layer	Male albino Wistar rats	(Karakurt et al. 2018)
*Redox (serum)*: ↑ MDA and MPO; ↓ tGSH and SOD *Inflammation (serum)*: ↑ TNF-α and IL-1β *Histological (retina)*: ↑ edema; ↑ degeneration; ↑ destruction; ↑ hemorrhage; ↑ thickness; ↑ astrocytes; ↑ polynuclear leucocytes	Rutin	*Redox (serum)*: ↓ MDA and MPO; ↑ tGSH and SOD *Inflammation (serum)*: ↓ TNF-α and IL-1β *Histological (retina)*: ↓ edema; ↓ degeneration; ↓ destruction; ↓ hemorrhage; ↓ thickness; ↓ astrocytes; ↓ polynuclear leucocytes	Male albino Wistar rats	(Taşlı and Uçak 2018)
*Redox (optic nerve tissues)*: ↑ MDA and TOS; ↓ tGSH and TAS *Inflammation (optic nerve tissues)*: ↑ NF-κB *Histological (optic nerve)*: ↑ edema, hemorrhage; ↑ degeneration	Taxifolin	*Redox (optic nerve tissues)*: ↓ MDA and TOS; ↑ tGSH and TAS *Inflammation (optic nerve tissues)*: ↓ NF-κB *Histological (optic nerve)*: ↓ edema, hemorrhage; ↓ degeneration	Male albino Wistar rats	(Ahiskali et al. 2021)
*Redox (optic nerve tissues)*: ↑ MDA, TOS, and OSI; ↓ tGSH and TAS *Inflammation (optic nerve tissues)*: ↑ TNF-α and NF-κB *Histological (optic nerve)*: ↑ dilated blood vessels; ↑ edema, hemorrhage; ↑ proliferating capillaries; ↑ degeneration	Resveratrol	*Redox (optic nerve tissues)*: ↓ MDA, TOS, and OSI; ↑ tGSH and TAS *Inflammation (optic nerve tissues)*: ↓ TNF-α and NF-κB *Histological (optic nerve)*: ↓ dilated blood vessels; ↓ edema, hemorrhage; ↓ proliferating capillaries; ↓ degeneration	Male albino Wistar rats	(Agcayazi et al. 2021)
*Redox (serum)*: ↑ MDA and TOS; ↓ tGSH and TAS *Histological (retina)*: ↑ edema; ↑ destruction; ↑ vascular congestion; ↑ polymorphonuclear cell; ↓ number of ganglion cells	Coenzyme Q10	*Redox (serum)*: ↓ MDA; ↓ TOS; ↑ tGSH and TAS *Histological (retina)*: ↓ edema; ↓ destruction; ↓ vascular congestion; ↓ polymorphonuclear cell; ↑ number of ganglion cells	Male Wistar albino rats	(Sunar et al. 2021)
*Redox (ocular tissue)*: – TOS and TAS *Apoptosis (cornea)*: ↑ apoptotic cells (TUNEL assay) *Proteostasis*: ↓ HSP70 (ciliary body); ↑ HSP90 (retina and ciliary body) Histological (retina): ↓ retinal thickness Histological (retina, cornea, ciliary body): ↑ edema; ↑ degeneration; ↑ disorganization	Melatonin	*Redox (ocular tissue)*: – TOS and TAS *Apoptosis marker (cornea)*: ↓ apoptotic cells (TUNEL assay) *Proteostasis (retina and ciliary body)*: ↑ HSP70 (retina and ciliary body); ↑ HSP90 (retina and ciliary body) Histological (retina): ↓ retinal thickness Histological (retina, cornea, ciliary body): ↓ edema; ↓ degeneration; ↓ disorganization (*Partial protection)	Wistar rats	(Polat et al. 2023)

↓Decreased ↑ Increased—No effect

**Table 2:** Effects of antioxidant intervention on cisplatin-induced cognitive impairment

Effect of cisplatin	Antioxidant	Protective effects	Model	References
*Development*: ↓ body weight; – brain weight *Behavior*: ↓ spatial learning and memory (Morris Water Maze); ↓ retention memory and spontaneous alternation (Y-Maze test) *Redox markers (brain homogenate)*: ↑ MDA and AChE; ↓ GPx, SOD, GSH, and CAT *Inflammation markers (brain homogenate)*: ↑ TNF-α, IL-6, and IL-1β *Apoptosis markers (brain tissue)*: ↓ Bcl-2; ↑ Caspase-3 and p53	Yanang *(Tiliacora triandra* Colebr. Diels) extract	*Development*: ↑ body weight; – brain weight *Behavior*: ↓ latency time to locate the hidden platform (Morris Water Maze); ↑ retention memory and spontaneous alternation (Y-Maze test) *Redox markers*: ↓ MDA and AChE; ↑ GPx, SOD, GSH, and CAT Inflammation markers ↓ TNF-α, IL-6, and IL-1β *Apoptosis*: ↑ Bcl-2; ↓ Caspase-3 and p53	Male Wistar Rats	(Huang et al. 2021)
*Behavior*: ↓ locomotor activity (Rotarod test); ↓ spatial learning and memory (Morris Water Maze) *Redox markers (hippocampus)*: ↑ MDA, NO, and XO; ↓ SOD, GPx, GST, CAT, GSH and TAC *Inflammation markers (hippocampus)*: ↑ TNF-α, IL-1β, IL-6, IL-12 *Amyloidogenic markers (hippocampus)*: ↓ Aβ-40; ↑ Aβ-42 *Neurotransmitter markers (hippocampus)*: ↓ ACh, NE, DA, and 5-HT; ↑ AChE and MAO *Apoptosis markers (hippocampus)*: ↑ p53 and Caspase-3 *Histological (hippocampus)*: ↑ neurodegeneration and pericellular vacuolation; ↑ dilated blood vessel; ↑ p53 and Bax	Ginseng *(Panax ginseng* C.A. Meyer) extract	*Behavior*: ↑ locomotor activity (Rotarod test); ↑ spatial learning and memory (Morris Water Maze) *Redox markers (hippocampus)*: ↓ MDA, NO, and XO; ↑ SOD, GPx, GST, CAT, GSH and TAC *Inflammation markers (hippocampus)*: ↓ TNF-α, IL-1β, IL-6, and IL-12 *Amyloidogenic markers (hippocampus)*: ↑ Aβ-40; ↓ Aβ-42 *Neurotransmitter markers (hippocampus)*: ↑ ACh, NE, DA, and 5-HT; ↓ AChE and MAO *Apoptosis markers (hippocampus)*: ↓ p53 and Caspase-3 *Histological (hippocampus)*: ↓ neurodegeneration and pericellular vacuolation; ↓ dilated blood vessel; ↓ p53 and Bax	Male Sprague–Dawley rats	(Hussien and Yousef 2022)
*Behavior*: ↓ cold sensitivity; ↓ heat sensitivity *Biochemical markers*: ↑ creatinine, urea, ALT, and AST; ↑ platelet and white blood cell; ↓ hemoglobin and red blood cell *Redox markers*: ↑ NO and MDA; ↓ CAT and GSH *Histological*: ↓ number of neurons in the cortex; ↑ GFAP; ↓ Bcl-2	Sea Urchin *(Diadema savignyi* Audouin) extract	*Behavior:* ↑ cold sensitivity; ↑ heat sensitivity *Biochemical markers*: ↓ creatinine, urea, ALT, and AST; ↓ platelet and white blood cell; ↑ hemoglobin and red blood cell *Redox markers*: ↓ NO and MDA; ↑ CAT and GSH *Histological*: ↑ number of neurons in the cortex; ↓ GFAP; ↑ Bcl-2	Male Sprague–Dawley rats	(Khalil et al. 2023)
*Behavior*: ↓ swimming response; ↑ immobility response; ↓ climbing time; ↓ sociability; ↓ memory performance (modified T-Maze test) *Redox markers*: ↓ GPx and SOD; ↑ MDA *Inflammation*: ↑ TNF-α and IL-1β; ↑ NLRP3, IL-6, and Nrf-2 *Apoptosis*: ↑ caspase-3 and BAX; ↓ BCL-2 *Histological (cortex and hippocampus)*: ↑ neuronal shrinkage, degeneration, and vacuolation; ↑ neurofibrillary tangles; ↑ caspase-3 and GFAP	*Nigella sativa* L. oil (NSO)	*Behavior*: ↑ swimming response; ↓ immobility response; ↑ climbing time; ↑ sociability; ↑ memory performance (modified T-Maze test) *Redox markers*: ↑ GPx and SOD; ↓ MDA *Apoptosis*: ↓ caspase-3 and BAX; ↑ BCL-2 *Inflammation*: ↓ TNF-α and IL-1β; ↓ NLRP3, IL-6, and Nrf-2 *Histological (cortex and hippocampus)*: ↓ neuronal shrinkage, degeneration, and vacuolation; ↓ neurofibrillary tangles; ↓ caspase-3 and GFAP	Male Wistar rats	(Elkattawy et al. 2026)
*Behavior*: ↑ freezing time (fear conditioning); ↓ discrimination ratio (context-object discrimination and novel object recognition) *Neuronal (hippocampus)*: ↑ apoptotic cells In vitro model *Oxidative stress/Apoptosis*: ↑ ROS; ↑ cleaved Caspase-9 *Neuronal development*: ↑ dendritic damage (spine density)	N-Acetylcysteine	*Behavior*: ↓ freezing time (fear conditioning); ↑ discrimination ratio (context-object discrimination and novel object recognition) *Neuronal (hippocampus)*: ↓ apoptotic cells In vitro model *Oxidative stress/apoptosis*: ↓ ROS; ↓ cleaved Caspase-9 *Neuronal development*: ↓ dendritic damage (spine density)	Male Sprague–Dawley rats; cultured hippocampal neurons and NSC	(Lomeli et al. 2017)
*Behavioral/Spatial/Cognitive (novel object recognition)*: ↓ total exploration; ↓ object exploration; ↓ discrimination ratio *Redox markers (hippocampus and frontal cortex)*: ↑ cleaved Caspase-9; ↓ GSH *Neuronal (hippocampus)*: ↑ dendritic damage	N-Acetylcysteine	*Behavior (novel object recognition)*: ↑ total exploration; ↑ object exploration; ↑ discrimination ratio *Redox markers (hippocampus and frontal cortex)*: ↑ GSH *Apoptosis*: ↓ cleaved Caspase-9 *Neuronal (hippocampus)*: ↓ dendritic damage	*Tumor-bearing model (Ovarian cancer xenograft model); primary hippocampal neurons*	(Lomeli et al. 2025)
↑ survival *Development*: ↓ weight *Tumoral development*: ↓ tumor volume	N-Acetylcysteine	↑ survival *Development*: ↓ weight *Tumoral development*: ↓ tumor volume	Tumor-bearing model (Ovarian cancer xenograft model)	(Lomeli et al. 2025)
*Behavior (Morris Water Maze)*: ↓ spatial learning and memory (Morris Water Maze) *Redox markers (hippocampus)*: ↑ MDA; ↓ thiol and SOD	Vitamin E	*Behavioral/Spatial/Cognitive (Morris Water Maze)*: ↑ spatial learning and memory (Morris Water Maze) *Redox markers (hippocampus)*: ↓ MDA; ↑ thiol and SOD	Male rats	(Hosseinzadeh et al. 2021)
*Behavior*: ↓ unfamiliar arm choice (T-Maze test); ↓ motor deficit (Rotarod test); – heat sensitivity *Biochemical (brain tissue)*: ↑ AChE and MAO *Redox markers (brain tissue)*: ↑ MDA; ↓ SOD and GPx; – GR *Inflammation markers (brain tissue)*: ↑ TNF-α, IL-1ß, and IL-6	Melatonin	*Behavior*: ↑ unfamiliar arm choice (T-Maze test); ↑ motor deficit (Rotarod test); – heat sensitivity *Biochemical markers (brain tissue)*: ↑ AChE; ↓ MAO *Redox markers (brain tissue)*: ↓ MDA; ↑ SOD and GPx; ↑ GR *Inflammation markers (brain tissue)*: ↑ TNF-α, IL-1ß, and IL-6	Male Sprague–Dawley rats	(Bayraktar et al. 2022)
Spatial memory deficits (novel location recognition test); reduced hippocampal dendritic spine density (CA3 region)	Melatonin	Prevented memory deficits; reversed dendritic spine density reduction	Male Wistar rats	(Qutifan et al. 2024)
*Survival and development*: ↑ mortality; ↓ body weight *Behavior*: ↓ spatial learning and memory (Morris Water Maze); – swimming speed (MORRIS Water Maze); ↓ recognition index (NOR test); – exploration time (NOR test); ↓ motor coordination (Rotarod test) *Biochemical markers (hippocampus)*: ↑ AChE *Redox markers (hippocampus)*: ↑ MDA,; ↓ SOD and CAT *Inflammation markers (hippocampus)*: ↑ IL-1β, TNF-α, and NF-κB; ↓ Nrf2 and HO-1 *Neuronal development (hippocampus)*: ↓ BDNF	Edaravone	*Survival and development*: ↓ mortality; ↑ body weight *Behavior*: ↑ spatial learning and memory (Morris Water Maze); – swimming speed (MORRIS Water Maze); ↑ recognition index (NOR test); – exploration time (NOR test); ↑ motor coordination (Rotarod test) *Biochemical markers (hippocampus)*: ↓ AChE *Redox markers (hippocampus)*: ↓ MDA; ↑ SOD and CAT *Inflammation markers (hippocampus)*: ↓ IL-1β and TNF-α; ↓ NF-κB; ↑ Nrf2 and HO-1 *Neuronal development (hippocampus)*: ↑ BDNF	Male Wistar rats	(Jangra et al. 2016)
In vitro *Cell metabolism*: ↓ viability In vivo *Behavior*: ↓ locomotor activity (digital actophotometer); ↓ muscle strength (Rotarod method); ↑ cognitive despair *Redox markers:* ↑ MDA; ↓ GSH and CAT *Histological (cerebellum)*: ↑ neuronal loss	Sitagliptin (drug repurposing)	In vitro *Cell metabolism*: ↑ viability In vivo *Behavior*: ↑ locomotor activity (digital actophotometer); ↑ muscle strength (Rotarod method); ↓ cognitive despair *Redox markers*: ↓ MDA; ↑ GSH and CAT *Histological (cerebellum)*: ↓ neuronal loss	PC12 cells; Male albino Wistar rats	(Li et al. 2019)
*Behavior*: ↓ spatial learning and memory (T-maze test) *Redox markers (hippocampus)*: ↑ MDA; ↓ SOD and GSH Mitochondrial metabolism/Apoptosis markers (hippocampus): ↓ PGC-1α; ↑ Caspase-3, CHOP, and GRP78 *Histological (hippocampus)*: ↑ neurodegeneration and vacuolization	Linagliptin (drug repurposing)	*Behavior*: ↑ spatial learning and memory Redox markers (hippocampus): ↓ MDA; ↑ SOD and GSH *Mitochondrial metabolism/Apoptosis markers (hippocampus)*: ↑ PGC-1α; ↓ Caspase-3, CHOP, and GRP78 *Histological (hippocampus)*: ↓ neurodegeneration and vacuolization	Male BALB/c mice	(El-Deeb et al. 2020)
*Development*: ↓ body weight *Behavior*: ↓ latency in entering the dark region (Passive Avoidance test); ↑ time spent in the darkroom (Passive Avoidance test); ↑ number entries in the darkroom (Passive Avoidance test); ↑ anxiety (Open Field test); ↑ thermal sensitivity; ↓ grip strength; ↓ SNCV, SNAP, and H-reflex amplitude (electrophysiological examinations); – MNCV, CMAP, H-reflex latency (electrophysiological examinations) *Redox markers*: ↓ SOD and GPx; ↑ MDA *Inflammation*: ↑ MMP-2 and MMP-9; ↑ TNF-α and IL-1β *Histological*: ↓ dorsal root ganglion type A cell; ↑ dorsal root ganglion type B cell; ↑ 4-HNE, TNF-α, and IL-1β	Mesna (thiol compound; drug repurposing)	*Development*: ↑ body weight *Behavior*: ↑ latency in entering the dark region (Passive Avoidance test); ↓ time spent in the darkroom (Passive Avoidance test); ↓ number entries in the darkroom (Passive Avoidance test); ↓ anxiety (Open Field test); ↓ thermal sensitivity; ↑ grip strength; ↑ SNCV, SNAP, and H-reflex amplitude (electrophysiological examinations); – MNCV, CMAP, H-reflex latency (electrophysiological examinations) *Redox markers*: ↑ SOD and GPx; ↓ MDA *Inflammation*: ↓ MMP-2 and MMP-9; ↓ TNF-α and IL-1β *Histological*: ↑ dorsal root ganglion type A cell; ↓ dorsal root ganglion type B cell; ↓ 4-HNE, TNF-α, and IL-1β	Male Wistar rats	(Saadati et al. 2021)
*Behavior (Morris Water Maze)*: ↑ swimming (distance; training); ↑ time learning to swimming *Redox markers/Transcriptional factors (cortex)*: ↓ p62, Pink1, Mtor; – Nfe2l2, Akt1, Bdnf; ↓ Gclc, Gpx, Prdx3, Txnr2, and SOD2 Redox markers/Transcriptional factors (hippocampus): ↓ Pink1, Nfe2l2, Akt1, Bdnf; – p62, Mtor; ↓ Gclc, Gpx, Prdx3, and SOD2; – Txnr2 *mt*DNA: ↑ in the cortex; ↓ in the hippocampus gut microbiome: dysbiosis	Methylene Blue and Azur B	*Behavioral/Spatial/Cognitive (Morris Water Maze)*: ↓ swimming (distance; training); ↓ time learning to swimming *Redox markers/Transcriptional factors (cortex)*: ↑ p62, Pink1, Mtor, Nfe2l2, Akt1, and Bdnf; ↓ Gclc, Gpx, Prdx3, Txnr2, and SOD2 *Redox markers/Transcriptional factors (hippocampus)*: ↑ p62, Mtor; ↓ Nfe2l2, Pink1, Akt1, and Bdnf; ↑ Gclc; ↑ Gpx, Prdx3, and SOD2; – Txnr2 *mt*DNA: ↓ in the cortex; ↑ in the hippocampus gut microbiome: prevented dysbiosis	C57BL/6 mice	(Krutskikh et al. 2022)
*Cell cycle/Metabolism markers*: ↑ cycle arrest; ↑ differentiation; ↓ self-renewal; ↑ glycolysis suppression *Oxidative stress marker*: ↑ ROS Mitochondria: ↑ activity (MTT assay), ATP, and Δψm	Mito-TEMPO (mitochondria-targeted antioxidant)	*Cell cycle/Metabolism markers*: ↓ S-phase entry; ↓ adherence; ↓ differentiation *Oxidative stress marker*: ↓ ROS	NSPCs	(Bustamante-Barrientos et al. 2025)
*Behavior (Passive Avoidance test)*: ↓ latency in entering the dark region; ↑ time spent in the darkroom *Biochemical marker (hippocampus, cortex, and cerebellum)*: ↑ AChE *Redox markers (hippocampus, cortex, and cerebellum)*: ↓ SOD and total thiol; ↑ MDA	Probiotics, Prebiotics, and Synbiotics	*Behavioral/Spatial/Cognitive (Passive Avoidance test)*: ↑ latency in entering the dark region; ↓ time spent in the darkroom Biochemical marker ↓ AChE *Oxidative stress markers*: ↑ SOD and total thiol; ↓ MDA	Female Wistar rats (premature ovarian failure model)	(Madahali et al. 2025)
*Behavior*: ↓ spontaneous alternation percentage (Y-Maze test); ↓ step-through latency (Passive Avoidance test); ↓ locomotion (locomotor activity detector); ↓ motor coordination (Rotarod test) Histological (hippocampus and cortex): ↑ apoptosis; ↑ neurodegeneration; ↑ MDA; ↓ CAT; ↑ GFAP, NF-κB, TNF-α, and IL-6	Captopril	*Behavior*: ↑ spontaneous alternation percentage (Y-Maze test); – passive avoidance test; ↑ step-through latency; ↑ locomotion; ↑ motor coordination *Histological (hippocampus and cortex)*: ↓ apoptosis; ↓ neurodegeneration; ↓ MDA; ↑ CAT; ↓ GFAP, NF-κB, TNF, and IL-6	Female Sprague–Dawley rats	(Mostafa et al. 2025)
*Survival and development*: ↑ mortality rate; ↓ body weight *Behavior*: – memory deficits (Y-maze) *Redox markers*: ↓ CAT and GPx-1; ↑ ROS; – SOD and MDA	Tirzepatide (negative study)	*Survival and development*: ↑ mortality rate; ↓ body weight Behavior: – memory deficits (Y-maze) Redox markers: – CAT and GPx-1; ↓ ROS levels; – SOD and MDA	Female albino Wistar rats	(Almutairi and Alhowail 2026)

↓Decreased ↑ Increased—No effect

**Table 3:** Effects of antioxidant intervention on cisplatin-induced peripheral neuropathy

Effect of cisplatin	Agent	Protective effects	Model	References
Sensory and motor deficits (rotarod, hot plate, cold plate, tail flick) *Biochemical (Serum and Sciatic nerve tissues)*: ↓ CAT, GPx1 and SOD2; ↑ MDA, TOS, IMA; ↑ NF-κB, TNF-α, IL-6	*Taraxacum officinale* leaf extract (rich in polyphenols: luteolin and quercetin)	Restored sensory and motor functions *Biochemical*: ↑ Antioxidant enzymes CAT, GPx1, SOD2; ↓ Oxidative stress markers MDA, TOS, IMA; ↓ Proinflammatory cytokines NF-κB, TNF-α, IL-6 Molecular docking: luteolin/quercetin bind to NF-κB1	Male Wistar albino mice	(Erdem et al. 2025)
In vitro: ↓ cell viability; ↓ neurite growth Oxidative stress/DNA damage markers: ↑ ROS; ↑ H2A.X *Apoptosis markers*: ↑ apoptosis; ↑ cleaved Caspase-3; ↑ p53 In vivo *Behavior*: ↑ mechanical allodynia (Von Frey test); ↓ thermal sensitivity (Hargreaves test) Inflammation markers: –TNF-α, IL-1β, and IL-6	*Trichosanthes kirilowii* extract (active compound: Cucurbitacin D)	*In vitro:* ↑ cell viability; ↑ neurite growth *Oxidative stress/DNA damage markers*: ↓ ROS; ↓ H2A.X *Apoptosis markers*: ↓ apoptosis; ↓ cleaved Caspase-3; ↓ p53 In vivo *Behavior*: ↓ mechanical allodynia (Von Frey test); ↑ thermal sensitivity (Hargreaves test) *Inflammation markers*: – TNF-α, IL-1β, and IL-6	PC12 cells; Male Sprague Dawley rats	(Kang et al. 2025)
In vitro*:* ↑ Morphological alterations; ↑ p38 MAPK phosphorylation and nuclear translocation in DRG neurons; ↑ Oxidative stress; ↑ Mitochondrial dysfunction; ↑ Cleaved caspase-3 In vivo*:* Mechanical and musculoskeletal hyperalgesia, cold sensitivity *Behavior*: ↑ mechanical allodynia (Von Frey test); ↓ thermal sensitivity (Hargreaves test) Inflammation markers: –TNF-α, IL-1β, and IL-6	Neflamapimod (p38 MAPK alpha inhibitor)	*In vitro:* Inhibited morphological alterations; Inhibited p38 MAPK phosphorylation; ↓ Oxidative stress, mitochondrial dysfunction and cleaved caspase-3; Protected neuronal integrity, prevented axonal damage *In vivo:* Improved mechanical/musculoskeletal hyperalgesia, cold sensitivity *Behavior*: ↓ mechanical allodynia (Von Frey test); ↑ thermal sensitivity (Hargreaves test) *Inflammation markers*: – TNF-α, IL-1β, and IL-6	Transgenic breast or prostate cancer mouse model (C3TAg); Wild-type healthy mice (FVB/N); Isolated DRG neurons	(Goel et al. 2025b, a)
*Behavior*: ↓ motor coordination (Rotarod test); ↓ thermal sensitivity (cold/hot plate); ↓ mechanical pain threshold (tail flick assessment and Von Frey test); ↓ nerve conduction velocity (electrophysiological experiments) *Redox marker (sciatic nerve tissue)*: ↓ GPX4 *Inflammation marker (sciatic nerve tissue)*: ↑ NF-κB *Histological (sciatic nerve tissue)*: ↑ myelin deterioration; ↑ axonal degeneration	Fisetin (flavonoid)	*Behavior*: ↑ motor coordination (Rotarod test); ↑ thermal sensitivity (cold/hot plate); ↑ mechanical pain threshold (tail flick assessment and Von Frey test); ↑ nerve conduction velocity (electrophysiological experiments) *Redox marker (sciatic nerve tissue)*: ↑ GPX4 Inflammation marker (sciatic nerve tissue): ↓ NF-κB *Histological (sciatic nerve tissue)*: ↓ myelin deterioration; ↓ axonal degeneration	Male Wistar rats	(Adiguzel et al. 2025)
↓ body weight *Behavior*: ↓ thermal sensitivity (tail flick test); ↓ motor coordination (Rotarod test); ↓ grip strength; ↓ suspension time (wire hang test); ↑ falls (beam walk test) *Redox*: ↑ MDA; ↓ GSH, SOD, and CAT *Histological (sciatic nerve tissue)*: ↑ axonal; ↑ myelin degeneration	*Alpinia calcarata* extract (contains phytosterol stigmasterol)	↑ body weight *Behavior*: ↑ thermal sensitivity (tail flick test); ↑ motor coordination (Rotarod test); ↑ grip strength; ↑ suspension time (wire hang test); ↓ falls (beam walk test) *Redox*: ↓ MDA; ↑ GSH, SOD, and CAT *Histological (sciatic nerve tissue)*: ↓ axonal; ↓ myelin degeneration	Male Wistar rats	(Dhavale et al. 2026)
*Behavior*: ↓ motor coordination (Rotarod test); ↓ sensitivity to pressure force (Randell-Sellitto test); ↓ thermal sensitivity (Hot plate); ↓ sensory and motor nerve conduction velocity (electrophysiological experiments) *Redox markers (sciatic nerve homogenates)*: ↑ MDA and NO; ↓ Nrf2 *Inflammation markers (sciatic nerve homogenates)*: ↑ NF-κB *Metabolism/cell fate markers (sciatic nerve homogenates)*: ↓ AMPK; ↑ mTOR and PI3K; ↓ ATP; ↑ ADP and NADPH Histological (sciatic nerve): ↑ axonal; ↑ myelin degeneration; ↑ iNOS	Trimetazidine (TRI)	*Behavior*: ↑ motor coordination (Rotarod test); ↑ sensitivity to pressure force (Randell-Sellitto test); ↑ thermal sensitivity (Hot plate); ↑ sensory and motor nerve conduction velocity (electrophysiological experiments) *Redox markers (sciatic nerve homogenates)*: ↓ MDA and NO; ↑ Nrf2 *Inflammation markers (sciatic nerve homogenates)*: ↓ NF-κB *Metabolism/cell fate markers (sciatic nerve homogenates)*: ↑ AMPK; ↓ mTOR and PI3K; ↑ ATP; ↓ ADP and NADPH *Histological (sciatic nerve)*: ↓ axonal; ↓ myelin degeneration; ↓ iNOS	Male Wistar rats	(Elbaset et al. 2024)
*Behavior*: ↓ spatial learning and memory (Morris Water Maze);—motor nerve conduction velocity; – muscle action potential; ↓ sensory nerve conduction velocity; ↓ sensory nerve action potentials *Neuronal development markers (prefrontal cortex homogenate)*: ↓ BDNF *Redox markers (prefrontal cortex homogenate)*: ↓ SOD, GPx; ↑ MDA *Inflammatory markers (prefrontal cortex homogenate)*: ↑ IL-1β, TNF-α *Mitochondria marker*: ↓ Δψm	Calcitriol	*Behavior*: ↑ spatial learning and memory (Morris Water Maze); – motor nerve conduction velocity; – muscle action potential; ↑ sensory nerve conduction velocity; ↑ sensory nerve action potentials Neuronal development markers (prefrontal cortex homogenate): ↓ BDNF *Redox markers (prefrontal cortex homogenate)*: ↑ SOD, GPx; ↓ MDA *Inflammatory markers (prefrontal cortex homogenate)*: ↓ IL-1β, TNF-α *Mitochondria marker*: ↑ Δψm	Male Wistar rats	(Niapour et al. 2024)
↑ DN4 score (neuropathic pain questionnaire) ↑ CIPNAT score (peripheral neuropathy assessment) ↑ pain perception (peripheral neuropathy symptoms)	Livergol: silymarin extract derived from the seeds of *Silybum marianum* (L.) Gaertn	↓ DN4 score (neuropathic pain questionnaire) ↓ CIPNAT score (peripheral neuropathy assessment) ↓ pain perception (peripheral neuropathy symptoms)	Double-blind study on 60 cancer patients who received cisplatin chemotherapy treatment	(Gholami et al. 2024b, a)
In vitro* model* *Cell survival*: ↓ cell viability In vivo* model* *Development*: ↓ body weight *Behavior*: ↑ mechanical allodynia; ↓ thermal sensibility (hot/cold plate); ↓ motor coordination (Rotarod test); ↓ motor coordination (walking tracks) *Histological (sciatic nerve and DRG)*: ↑ axonal degeneration; ↑ TNF-α	Chlorogenic acid	In vitro* model* *Cell survival*: ↑ cell viability In vivo* model* *Development*: ↓ body weight *Behavior*: ↓ mechanical allodynia; ↑ thermal viability (hot/cold plate); ↑ motor coordination (Rotarod test); ↑ motor coordination (walking tracks) *Histological (sciatic nerve and DRG)*: ↓ axonal degeneration; ↓ TNF-α	Male Sprague–Dawley rats; Primary DRG cell culture	(Unel et al. 2024)
*Behavior*: ↑ nociceptive impairment (tail immersion test) *Biochemical markers (serum)*: ↓ NGF *Metabolism/cell fate markers (serum)*: ↓ mTOR *Histological (sciatic nerve)*: ↓ GSH; ↑ MDA; ↑ Caspase-3; ↑ axonal degeneration; ↑ myelin deterioration; ↑ LC3-II	Quercetin	*Behavior*: ↓ nociceptive impairment (tail immersion test) *Biochemical markers (serum)*: ↑ NGF *Metabolism/cell fate markers (serum)*: ↑ mTOR *Histological (sciatic nerve)*: ↑ GSH; ↓ MDA; ↓ Caspase-3; ↓ axonal degeneration; ↓ myelin deterioration; ↓ LC3-II	Male Wistar rats	(Mahmoud et al. 2023)
*Behavior*: ↓ thermal sensitivity (hot plate test) *Redox markers (DRG and sciatic nerve)*: ↑ MDA; ↓ SOD activity; – Nrf2, *Hmox1*, and *Gclm* *Apoptosis markers (DRG and sciatic nerve)*: ↑ p53 and bax/bcl2 ratio	1,3-Dimethylthiourea (DMTU)	*Behavior*: ↑ thermal sensitivity (hot plate test) *Redox (DRG and sciatic nerve)*: ↓ MDA; ↑ SOD activity; – Nrf2, *Hmox1*, and *Gclm* *Apoptosis (DRG and sciatic nerve)*: ↓ p53 and bax/bcl2 ratio	Male Sprague–Dawley rats	(Seto et al. 2023)
*Behavior*: ↓ compound muscle action potential (CMAP) amplitude (electrophysiological test); ↓ motor coordination (inclined plane score) *Redox markers (plasma)*: ↑ MDA; ↓ GSH *Inflammation markers (plasma)*: ↑ TNF-α and IL-6 *Proteostasis (sciatic nerve)*: ↓ HSP-70 *Histological*: ↓ axon diameter; ↓ NGF	Propofol	*Behavior*: ↑ compound muscle action potential (CMAP) amplitude (electrophysiological test); ↑ motor coordination (inclined plane score) *Redox markers (plasma)*: ↓ MDA; ↑ GSH *Inflammation markers (plasma)*: ↑ TNF-α and IL-6 *Proteostasis (sciatic nerve)*: ↑ HSP-70 *Histological*: ↑ axon diameter; ↑ NGF	Female Wistar rats	(Gonullu et al. 2023)
*Development*: ↓ Body weight *Behavior*: ↑ nociceptive impairment (tail-flick test) *Redox markers (plasma)*: ↑ MDA; – TAC *Inflammation markers (plasma)*: ↑ IL-1β, TNF-α	Dexamethasone and Citicoline	*Development*: ↑ Body weight *Behavior*: ↓ nociceptive impairment (tail-flick test) *Redox markers (plasma)*: ↓ MDA; ↑ TAC *Inflammation markers (plasma)*: ↓ IL-1β, TNF-α	Male mice	(Masoud et al. 2022)
*Redox markers (nerve tissue and sciatic nerve)*: ↑ MDA, MPO; ↓ tGSH, SOD *Histological (nerve tissue)*: ↑ swollen myelinated nerve fibers; ↑ myelin sheath degeneration	Agomelatine (Melatonin analog)	*Redox markers (nerve tissue and sciatic nerve)*: ↓ MDA, MPO; ↑ tGSH, SOD *Histological*: ↓ swollen myelinated nerve fibers; ↓ myelin sheath degeneration	Male Wistar albino rats	(Yucetas et al. 2019)
*Behavior*: ↑ mechanical and cold hyperalgesia; ↓ electrical thresholds of Aδ and C fibers *Oxidative stress marker*: ↑ ROS in DRG *Mitochondria marker:* ↑ depolarization-evoked Ca^2+^ transients in DRG neurons	Pioglitazone (PPARγ agonist, antidiabetic drug)	*Behavior*: ↓ mechanical and cold hyperalgesia; ↑ electrical thresholds of Aδ and C fibers *Oxidative stress*: ↓ ROS in DRG *Mitochondria*: ↓ depolarization-evoked Ca^2^⁺ transients in DRG neurons	Mouse model of cisplatin-induced hyperalgesia; dissociated mouse DRG neurons in vitro	(Khasabova et al. 2019)

↓Decreased ↑ Increased—No effect

**Table 4:** Effects of antioxidant Intervention on cisplatin-induced anxiety and depression

Effect of cisplatin	Agent	Protective effects	Model	References
*Behavioral/Emotional*: ↑ anxiety-like behavior (Open Field, Elevated Plus Maze) *Biochemical (hippocampus)*: ↑ lipid peroxidation, ↓ antioxidant enzymes (SOD, CAT, GPx), ↑ pro-apoptotic markers	N-Acetylcysteine (NAC)	*Behavioral/Emotional*: ↓ anxiety-like behavior *Biochemical (hippocampus)*: ↓ oxidative stress, ↓ apoptosis, restored antioxidant enzyme activity	Male Wistar rats	(Vukovic et al. 2019)
*Behavioral/Emotional*: ↑ anxiety-like behavior (Open Field, Elevated Plus Maze) *Biochemical (hippocampus)*: ↑ lipid peroxidation, ↓ antioxidant defenses, ↑ pro-apoptotic markers	*Satureja hortensis* L. extract (SH)	*Behavioral/Emotional*: ↓ anxiety-like behavior (with 100 mg/kg dose) *Biochemical (hippocampus)*: ↓ oxidative stress, restored antioxidant and anti-apoptotic activity	Male Wistar rats	(Kumburovic et al. 2019)
*Behavioral/Emotional*: cerebellar neurotoxicity, motor dysfunction, and anxiety *Biochemical (cerebellum)*: ↑ oxidative stress (MDA), ↓ antioxidants (GSH, SOD, CAT), ↑ inflammation (TNF-α, IL-1β, IL-6, MPO), ↑ TLR4/NF-κB signaling, ↑ apoptosis (caspase-3) *Histological*: Cerebellar damage	Daflon (micronized purified flavonoid fraction)	*Behavioral/Emotional*: ↓ anxiety-like behavior, improved motor dysfunction *Biochemical (cerebellum)*: ↓ oxidative stress, ↓ inflammation, ↓ TLR4/NF-κB signaling, ↓ apoptosis *Histological*: Preserved cerebellar structure	Male Wistar rats	(Fidelis et al. 2025)
*Behavioral/Emotional*: ↑ anxiety-like and depressive-like behaviors, ↓ memory performance (T-maze) *Biochemical (brain)*: ↑ oxidative stress (MDA), ↓ antioxidants (GPx, SOD, Nrf2), ↑ inflammation (TNF-α, IL-1β, IL-6, NLRP3), ↑ apoptosis (caspase-3, BAX/BCL2) *Histological*: Neuronal degeneration in cortex and hippocampus	*Nigella sativa* oil (NSO)	*Behavioral/Emotional*: ↓ anxiety and depressive-like behaviors, ↑ memory performance *Biochemical (brain)*: ↓ oxidative stress, ↓ inflammation (↓NLRP3), ↓ apoptosis, ↑ Nrf2/HO-1 pathway *Histological*: Reduced neurodegeneration	Male Wistar rats	(Elkattawy et al. 2026)
*Behavioral/Emotional*: ↑ anxiety and depressive-like behaviors *Sensory/Motor*: Mechanical and cold allodynia, muscle strength deficits, weight loss *Biochemical (DRG, PFC)*: ↑ inflammation, ↑ oxidative stress	Duloxetine + Hydrogen-Rich Water	*Behavioral/Emotional*: ↓ anxiety and depressive-like behaviors *Sensory/Motor*: Prevented allodynia and muscle deficits *Biochemical (DRG, PFC)*: ↓ inflammation, ↓ oxidative stress	Male and Female C57BL/6 mice	(Martínez-Martel et al. 2025b, a)
*Behavioral/Emotional*: ↑ anxiety and depressive-like behaviors *Sensory/Motor*: Tactile and cold allodynia, muscle strength deficits, weight loss *Biochemical (DRG, PFC)*: ↑ inflammation, ↑ oxidative stress	Hydrogen-Rich Water (HRW)	*Behavioral/Emotional*: ↓ anxiety and depressive-like behaviors *Sensory/Motor*: Prevented allodynia and functional deficits *Biochemical (DRG, PFC)*: ↓ inflammation, ↓ oxidative stress	Male and Female C57BL/6 mice	(Martínez-Martel and Pol 2023)
*Behavioral/Emotional*: ↑ anxiety-like behavior (Open Field), ↓ passive avoidance memory *Sensory/Motor*: Altered thermal sensitivity, ↓ muscle strength, peripheral sensory neuropathy *Biochemical (DRG, nerve)*: ↑ oxidative stress (MDA, 4-HNE), ↓ antioxidants (SOD, GPx), ↑ inflammation (TNF-α, IL-1β, MMP-2/9) *Histological (DRG)*: Morphological alterations	Mesna (Thiol Compound)	*Behavioral/Emotional*: ↓ anxiety-like behavior, improved memory *Sensory/Motor*: Restored thermal sensitivity and muscle strength, improved nerve conduction velocity *Biochemical (DRG, nerve)*: ↓ oxidative stress, ↓ inflammation. Histological (DRG): Preserved morphology	Male Wistar rats	(Saadati et al. 2021)

↓Decreased ↑ Increased—No effect

### Retinal/optic nerve toxicity

Cisplatin-induced toxic optic neuropathy is a rare, but severe condition (Bee et al. 2024). The photoreceptors in the retina are rich in polyunsaturated fatty acids and consequently require elevated oxygen levels, which makes the retina very susceptible to oxidative stress (Ibrahim et al. 2019). Cisplatin causes direct oxidative damage to optic nerve and retinal ganglion cells, leading to chronic subclinical degeneration that may become clinically relevant over time, manifesting as conditions such as glaucoma, for instance (Dulz et al. 2017). The retina is a very specialized neural tissue highly vulnerable to oxidative stress due to a combination of factors: the limited regenerative capacity of retinal ganglion cells, its exceptionally high oxygen and energy demands, and a dense population of mitochondria (Eldemerdash et al. 2025). Retinopathy damage is typically reversible, while optic nerve-damage tends to be irreversible (Bee et al. 2024).

Tempol, coenzyme Q10, lutein, polyphenols and flavonoids (rutin, hesperidin, taxifolin, resveratrol, pycnogenol) have been investigated as protective agents against cisplatin-induced optic damage. The radical-scavenging properties of these compounds makes them potent antioxidants capable of inhibiting the onset of oxidative stress-related events. By modulating the redox balance and related cellular pathways, they protected retina, optical nerve or both against the oxidative damage induced by cisplatin in animal models (Table 1).

A study in Wistar rats demonstrated that Tempol, a membrane-permeable nitrosative antioxidant, protected against the retinal degeneration by reducing oxidative stress, restoring autophagy flux, alleviating ER stress, enhancing NGF expression; therefore, preserving retinal ultrastructure (Eldemerdash et al. 2025). The antioxidant mechanism of Tempol is similar to that of the enzyme superoxide dismutase (SOD), which scavenges free radicals, inhibits the Fenton reaction and the oxidative stress in various preclinical models (Ham and Raju 2017).

Lutein is a non- provitamin A carotenoid found in vegetables (corn, spinach, broccoli, carrots), fish and eggs. Lutein directly scavenges free radicals, especially singlet oxygen (^1^O_2_). For this reason, studies have associated lutein with the prevention of age-related macular degeneration (Krinsky et al. 2003; Landrum and Bone 2001; Żółnowska et al. 2024). Due to its structure, lutein localizes in lipid-bilayer portion of membranes of the retina and inhibits the oxidative damage. On the other hand, its structure decreases its bioavailability; therefore, liposome-based carriers might be alternatives for the therapeutic administration of lutein (Kumar et al. 2024). Accordingly, the study by (Ibrahim et al. 2019) shows a superior protection of liposomal lutein in comparison with free lutein. Authors state that encapsulation of lutein into nanoparticle formulations (liposomal form) could change the biodistribution; increase the antioxidative property and the penetration of the drug. Although authors refer to the potent antioxidant property of lutein, they do not perform any assay to access this parameter, which would support their conclusion that the liposomal formulation increases the antioxidant property of lutein.

A study with the angiotensin II type 1 receptor blocker, azilsartan, demonstrated that the neuroprotection against cisplatin-induced retinal and optic nerve toxicity was associated with reduced levels of TNF-α, NF-kB and Caspase-3, but oxidative stress markers were not assessed (Raheem and Mohammed Ali Mahmood 2023). However, other studies have already reported the antioxidant effects of azilsartan in other tissues. It has been reported that angiotensin-II binding to the AT1 receptor induces oxidative stress and that azilsartan inhibits this event by blocking AT1 receptor (Al-Chlaihawi and Janabi 2023). Additionally, a study demonstrated that azilsartan inhibits NADPH oxidase in the vasculature; this enzyme generates free radicals and increases oxidative stress (Nguyen Dinh Cat et al. 2013). Another study reported that azilsartan significantly inhibited reactive oxygen species (ROS) generation, lipid peroxidation and preserved mitochondrial function, thereby protecting endothelial cells against oxidative damage (Liu et al. 2016). Therefore, the reduction of oxidative stress by azilsartan might have contributed to protect retina/optic nerve against cisplatin neurotoxicity in the referred study (Raheem and Mohammed Ali Mahmood 2023).

### Cognitive impairment (chemobrain)

In recent years, the neurotoxicity of cisplatin in the CNS, particularly the cognitive impairment known as “chemobrain,” has gained significant attention. Historically, research focused on peripheral neuropathy, as CNS toxicity was considered rare. However, studies from the last decade have demonstrated the importance of cisplatin toxicity in the CNS. The number of reports of cisplatin-induced cognitive impairment has increased considerably in recent years. Furthermore, the mechanisms by which a hydrophilic molecule like cisplatin can readily cross the blood–brain barrier (BBB) are now much better understood. Many conditions can disrupt the integrity of the BBB and facilitate the permeation of hydrophilic molecules. Chemotherapeutic agents that cross the BBB can directly induce oxidative stress and DNA damage in the brain; they can also cause CNS toxicity indirectly by inducing the release of pro-inflammatory cytokines, which in turn trigger oxidative stress and mitochondrial dysfunction in the brain (Otto-Dobos et al. 2024). Additionally, cytokines can disrupt the integrity of the BBB, thereby facilitating the penetration of hydrophilic molecules, such as cisplatin. There is evidence that cisplatin treatment induces persistent disruption of BBB integrity, which contributes to oxidative stress in the CNS (Patai et al. 2025; Ren et al. 2017).

Chemobrain or chemotherapy-induced cognitive impairment (including that induced by cisplatin) affects the hippocampus and it impairs learning, memory, speech, concentrating and processing speed (Sahu et al. 2021). Although the mechanism by which cisplatin induces cognitive impairment is not completely understood, oxidative stress has been implicated in the toxic mechanism (Alhowail 2025). Multiple studies published in the last decade converge to oxidative stress and mitochondrial dysfunction as central mechanisms in cisplatin-induced cognitive impairment (Table 2). The different antioxidants studied were able not only to ameliorate the redox state by decreasing ROS generation and increasing the antioxidant defense system, but also to interfere with other toxic mechanisms elicited by cisplatin. The restoration of redox balance is demonstrated by reduced lipid peroxidation (MDA, TBARS), restored antioxidant defense system: SOD, CAT, GPx, GSH and thiols (Hosseinzadeh et al. 2021; Huang et al. 2021; Li et al. 2019). Studies also demonstrated mitochondrial protection through reduced mitochondrial ROS production (Bustamante-Barrientos et al. 2025) and enhanced mitochondrial bioenergetics (Kondaveeti and Gupta 2026). Neuroinflammation and mitochondrial oxidative stress have been proposed as interconnected mechanisms in several neurological disorders as well as in cisplatin-induced neurotoxicity (Alhowail 2025; Mani et al. 2025). Accordingly, studies have demonstrated that antioxidants were able to inhibit inflammatory pathways: NF-κB pathway, pro-inflammatory cytokines (TNF-α, IL-1β, IL-6), microglial/astrocyte activation (reduced GFAP) and NLRP3 inflammasome (Elkattawy et al. 2026; Jangra et al. 2016; Mostafa et al. 2025), which confirms the link between ROS accumulation and neuroinflammation. It was also consistently demonstrated that antioxidants protected against apoptosis by reducing caspase-3 activation, modulating pro- and anti-apoptotic proteins (Bax/Bcl2 ratio) and decreasing p53 expression (Elkattawy et al. 2026; Huang et al. 2021; Lomeli et al. 2017). A study with Tirzepatide reported negative results (Almutairi and Alhowail 2026). Tirzepatide is a GLP-1 agonist that was found to improve spatial learning and memory in diabetic rats (Guo et al. 2023). Although TIRZ diminished ROS levels, it did not ameliorate the reduction in enzymatic antioxidants, indicating only a partial reduction of oxidative stress. Accordingly, it did not ameliorate memory deficits nor improved cognitive function (Almutairi and Alhowail 2026). This study confirms the crucial role of oxidative stress in both cisplatin-induced cognitive impairment and as a target for neuroprotective strategies.

A study showed that methylene blue and azur B prevented learning impairment in mice (Krutskikh et al. 2022). Although methylene blue and azur B are not direct antioxidants such as free radicals’ scavengers, they diminish oxidative stress indirectly. Methylene blue and azur B activate the Nrf2/ARE signaling pathway, thereby activating the cellular antioxidant defense and protecting against oxidative stress damage (Loboda et al. 2016). Sitagliptin and Linagliptin are dipeptidyl peptidase-4 (DPP-4) inhibitors clinically used to improve glycemic control in patients with type 2 diabetes mellitus. They also modulate the Nrf2 pathway thereby improving cellular antioxidant defense and protecting against oxidative stress damage (Civantos et al. 2017; Si et al. 2019). Accordingly, Li et al. (2019) demonstrated that sitagliptin increased antioxidant enzymes (superoxide dismutase, SOD and catalase, CAT), and reduced malondialdehyde (MDA), a marker of oxidative damage. Similar results were obtained with linagliptin (El-Deeb et al. 2020). These effects were associated with improved behavioral parameters related to motor and cognitive functions. Melatonin also activates NRF2 pathway (Ahmadi and Ashrafizadeh 2020); however, is it is also a potent free radical scavenger and its metabolites can act in sequence or together to neutralize ROS, forming a cascade of elimination of free radicals (Monteiro et al. 2024). Accordingly, recent studies in rats treated with melatonin and cisplatin have associated decreased oxidative stress in the brain with amelioration of spatial memory deficits, reversal of dendritic spines density reduction and improved behavioral T-Maze and Rotarod tests (Bayraktar et al. 2022; Qutifan et al. 2024).

Neuroprotection studies are frequently conducted in tumor-free animal models, but it is crucial to perform the studies in tumor-bearing animals to investigate whether the antioxidant therapy interferes with the antitumor activity of cisplatin. In our search, only one study using a tumor-bearing model was identified, and the antioxidant investigated was N-acetylcysteine (NAC). An interesting observation was that tumor-bearing rats, which received only vehicle (saline) also presented cognitive deficits. This result is consistent with clinical evidence showing that cancer patients can have cognitive impairments due to the disease itself, even before receiving treatment. In this study, delayed NAC protected against cisplatin-induced cognitive impairment, and moreover, without reducing the anti-tumor efficacy of cisplatin. Interestingly, NAC did not reverse the cognitive impairment observed in vehicle-treated animals. According to authors, this finding suggests that the mechanism elicited by tumor might not involve oxidative stress, differently from that of cisplatin neurotoxicity. They even suggest a mechanism involving cytokine dysregulation (Lomeli et al. 2025). However, it could also be attributed to the elapsed time between tumor implantation and NAC treatment, i.e., the timing of NAC intervention might have been too late to reverse the damage induced by the tumor. This implies that NAC treatment should occur not very long after cisplatin treatment, otherwise it would not be able to counteract toxicity of cisplatin as well.

### Peripheral neuropathy

Cisplatin causes a sensory proprioceptive peripheral neuropathy (Mohammad et al. 2018), i.e., it targets the axons and neurons in the dorsal root ganglia (DRG), resulting in a progressive neuropathy that might persist even after chemotherapy cessation (Goel and Argueta 2025). The dorsal root ganglia (DRG) are particularly vulnerable to reactive oxygen species (ROS)-induced damage, as the blood–nerve and perineurial barriers are weak as compared to the BBB. ROS damages mitochondrial membrane integrity and mt-DNA, resulting in mitochondrial dysfunction. ROS also activate the mitogen-activated protein kinase (MAPK) pathway. Mitochondrial dysfunction and MAPK signaling converge to apoptosis (Ding et al. 2017; Kahya et al. 2017; Ott et al. 2007).

Table 3 presents a diverse range of antioxidant compounds, from plant extracts to repurposed drugs that protect against the oxidative stress and neuroinflammation induced by cisplatin mainly in rats and mice. These antioxidants act by different mechanisms of action, that might be directly by scavenging free radicals, or indirectly through activation of the antioxidant NRF2 pathway, which induces the expression of antioxidant defense enzymes. Upon exposure to oxidative stress, the nuclear factor erythroid 2-related factor 2 (NRF2) is activated and translocates into the nucleus, where it binds to the antioxidant response element (ARE), thereby promoting the transcription of antioxidant enzymes (Dong et al. 2008; Tchounwou et al. 2021). NRF2 functions as a key sensor of oxidative stress and has been implicated in the mechanism of neuroprotection of several natural products including silymarin (García-Muñoz and Victoria-Montesinos 2024). Silymarin also scavenges free radicals (Iraqi et al. 2025) and chelates transition metals such as iron (Fe), thereby preventing the formation of hydroxyl radicals via Fenton reaction. Moreover, silymarin inhibits the activity of pro-oxidant enzymes (García-Muñoz and Victoria-Montesinos 2024). Silymarin is extracted from Milk thistle (*Silybum marianum* L.), a wild plant commonly used in traditional medicine to treat liver diseases (Iraqi et al. 2025). A double-blind randomized clinical trial (RCT) study with Silybum Marianum (SM), performed in 60 cancer patients treated with cisplatin, showed that SM diminished the scales DN4 and CIPNAT in comparison with the placebo group. DN4 and CIPNAT are both assessment tools (scales) used to evaluate peripheral neuropathy. DN4 (Douleur Neuropathique, french) is a questionnaire used to differentiate neuropathic pain from nociceptive pain; CIPNAT (Chemotherapy-Induced Peripheral Neuropathy Assessment Tool) assesses the severity, nature, and impact of chemotherapy-induced peripheral neuropathy symptoms on daily life. In this study, authors attributed the positive results of SM to its potent antioxidant properties (Gholami et al. 2024b, a).

A study with neflamapimod (a p38 MAPK alpha inhibitor) used both tumor-bearing animals (C3TAg transgenic mice) and healthy mice to differentiate between cancer pain and chemotherapy-induced pain (Goel et al. 2025b, a). However, the study did not examine whether the treatment interferes with the anti-tumor activity of cisplatin, despite this being a critical consideration for future clinical trials.

### Anxiety/depression

Cerebellum is very susceptible to cisplatin-induced oxidative damage, particularly cerebellar cortex and Purkinje neurons. Cisplatin causes ROS generation, depletes the antioxidant capacity of cerebellar cells, leading to cerebellar oxidative damage. Cisplatin also activates inflammatory signaling pathways that, in turn, induces oxidative stress and amplifies cerebellar damage (Mohsen et al. 2019; Mokhtar et al. 2022; Moreno-Rius 2018). The cerebellum plays a role in motor function, fear and anxiety (Moreno-Rius 2018); it is also associated with spatial cognition, sensorimotor memory and language (Hadjiosif et al. 2024). The studies presented in Table 4 correlate the cerebellar damage induced by cisplatin with anxiety/depressive-like behaviors, impairments in locomotor activity and memory through neurobehavioral tests such as the Elevated Plus Maze, Open Field Test and T-Maze. These neurobehavioral alterations are mostly associated with oxidative stress (increased lipid peroxidation and reduced activity of antioxidant enzymes SOD, CAT, and GPx), neuroinflammation (upregulation of TNF-α, IL-1β, IL-6, and NLRP3), and activation of apoptotic pathways (increased caspase-3 and BAX/BCL2 ratio) in the hippocampus, prefrontal cortex, and/or cerebellum. The study by Fidelis et al. (2025) demonstrates that cisplatin-induced cerebellar neurotoxicity and anxiety-like behavior are mediated by the TLR4/NF-κB signaling pathway. The relationship between toll-like receptor 4 (TLR4) and oxidative stress is central to this neurotoxic mechanism of cisplatin. The tissue damage caused by cisplatin induces the release of damage-associated molecular patterns (DAMPs), which activate TLR4 signaling. TLR4 signaling promotes the production of reactive oxygen species (ROS) via NADPH oxidase (NOX) enzymes and mitochondrial dysfunction, and the resulting oxidative stress causes further cellular damage, releasing more DAMPs that re-activate TLR4. This amplification loop sustains neuroinflammation and neuronal injury, which is associated with the neurobehavioral deficits observed (Katanić Stanković et al. 2023). Several agents with antioxidant and anti-inflammatory properties have been shown to attenuate these neurotoxic effects of cisplatin in rats and mice. These include classical antioxidants such as N-acetylcysteine or NAC (Katanić Stanković et al. 2023); plant extracts/oil like *Satureja hortensis* (Kumburovic et al. 2019) and *Nigella sativa* oil (Elkattawy et al. 2026); flavonoid, like daflon (Fidelis et al. 2025); the chemoprotective drug mesna (Saadati et al. 2021) and alternative approaches such as hydrogen-rich water (HRW), either alone or in combination with the antidepressant duloxetine (Martínez-Martel et al. 2025b, a; Martínez-Martel and Pol 2023). 2-Mercaptoethane sulfonate (mesna) is a synthetic compound approved by the FDA in 1988 as a cytoprotective agent. It is used clinically to prevent the urotoxicity associated with cyclophosphamide and ifosfamide chemotherapy. (Vieira et al. 2003). Mesna is a thiol compound that scavenges reactive oxygen species (ROS) thereby preventing apoptotic cell death and alleviating the side effects of the chemotherapeutic agents (Dolgun et al. 2010; Jost et al. 2017; Li et al. 2013). This study (Saadati et al. 2021) demonstrates that, not only anxiety, but also cognitive and motor deficits induced by cisplatin are improved by mesna treatment.

Hydrogen-rich water (HRW) readily crosses the blood–brain barrier and cell membranes. It contains molecular hydrogen, which reduces the effects of oxidative stress by inhibiting excessive ROS production and activating the Nrf2 antioxidant transcription factor. It also reduces inflammation by blocking NF-κB and the NLRP3 inflammasome. (Chen et al. 2021; LeBaron et al. 2019). According to authors, HRW reduced anxiogenic and depressant behaviors in mice due to the reduction of oxidative/inflammatory damage (Martínez-Martel and Pol 2023).

In summary, the protective effects of all these different interventions presented in Table 4 are attributed to the restoration of redox balance, inhibition of inflammatory mediators and suppression of apoptotic markers. In the case of daflon, an additional pathway is suggested, i.e., the downregulation of the TLR4/NF-κB pathway, which exacerbates oxidative stress and neuroinflammation.

The majority of preclinical research focuses on cognitive and peripheral nerve function. The quantity of studies exploring emotional alterations such as anxiety-like and depressive-like behavior is markedly lower. Typically, anxiety or depressive parameters are assessed marginally into broader experimental designs primarily aimed at evaluating cognitive or peripheral nerve function, rather than being the central focus of the study.

## Conclusion

The neurotoxicity of cisplatin is an important limiting factor in cisplatin chemotherapy. It results from an interplay of events in which oxidative stress initiates a cascade involving mitochondrial dysfunction, ER stress, DNA damage, neuroinflammation and apoptosis. Mitochondria are both the primary site of ROS formation and a critical target of the oxidative damage, that amplifies ROS production, triggers the activation of caspases and leads to neuronal apoptosis. Mitochondrial impairment and oxidative stress induce microglial activation and initiate neuroinflammation. On the other hand, inflammatory cells increase ROS formation, intensifying the damage to peripheral and central nervous system. Antioxidant intervention has remained the primary and most promising strategy emerging from pre-clinical research over the last decade. In recent years, neurotoxicity induced in the central nervous system has gained more attention. In the past, it was considered rare; however, it has become clear that conditions affecting the blood–brain barrier and allowing the penetration of cisplatin into the CNS are more frequent than previously thought. Cisplatin neurotoxicity manifests mainly as: cognitive impairment, peripheral neuropathy, anxiety, depression, and retinopathy. Studies have demonstrated that a wide variety of agents, from natural compounds to repurposed drugs, can effectively counteract these effects. Their mechanisms include directly scavenging free radicals and/or indirectly inducing endogenous antioxidant defenses through the NRF2 pathway. By restoring redox balance, these interventions successfully reduce inflammation and prevent neuronal damage in animal models. Despite these promising pre-clinical results, they have not advanced into clinical trials. One contributing factor to this gap is the lack of studies in tumor-bearing animals. There is the concern that these neuroprotective antioxidants might interfere with the anti-tumor activity of cisplatin. To confirm the safety and efficacy of these strategies, future pre-clinical studies should be performed in tumor-bearing animal models.

## Declarations

### Confl ict of Interest

Authors declare that they have no confl icts of interest.
